# Development of a dyspnoea word cue set for studies of emotional processing in COPD

**DOI:** 10.1016/j.resp.2015.12.006

**Published:** 2016-03

**Authors:** Mari Herigstad, Anja Hayen, Andrea Reinecke, Kyle T.S. Pattinson

**Affiliations:** a FMRIB Centre, Nuffield Department of Clinical Neurosciences, University of Oxford, Oxford, UK; b Clinical Health Care, Oxford Brookes University, UK; c Department of Psychology, University of Reading, UK; d Department of Psychiatry, University of Oxford, UK

**Keywords:** CES-D, center for epidemiological studies depression questionnaire, COPD, chronic obstructive pulmonary disease, FMRI, functional magnetic resonance imaging, MRI, magnetic resonance imaging, SD, standard deviation, SGRQ, St. George respiratory questionnaire, STAI, state and trait anxiety inventory, VAS, visual analogue scale, Dyspnoea, COPD, Anxiety, Method

## Abstract

Patients with chronic dyspnoea may learn to fear situations that cue dyspnoea onset. Such dyspnoea-specific cues may then cause anxiety, and worsen or trigger dyspnoea even before commencement of physical activity. We therefore developed an experimental tool to probe emotional processing of dyspnoea for use with neuroimaging in COPD. The tool consists of a computerised task comprising multiple presentations of dyspnoea-related word cues with subsequent rating of dyspnoea and dyspnoea-anxiety with a visual analogue scale. Following 3 development stages, sensitivity to clinical change was tested in 34 COPD patients undergoing pulmonary rehabilitation. We measured internal consistency, sensitivity to clinical change and convergence with established dyspnoea measures (including Dyspnoea-12). Cronbach’s alpha was 0.90 for dyspnoea and 0.94 for dyspnea-anxiety ratings. Ratings correlated with Dyspnoea-12 (dyspnoea: *r* = 0.51, *P* = 0.002; dyspnea-anxiety: *r* = 0.54, *P* = 0.001). Reductions in dyspnea-anxiety ratings following pulmonary rehabilitation correlated with reductions in Dyspnoea-12 (*r* = 0.51, *P* = 0.002). We conclude that the word-cue task is reliable, and is thus a potentially useful tool for neuroimaging dyspnoea research.

## Highlights

• The first cue-based task to explore recall of dyspnoea and dyspnoea-related anxiety in COPD.
• Patients’ dyspnoea and dyspnoea-anxiety ratings agreed with established measures of dyspnoea.
• Patients’ dyspnea-anxiety ratings changed in accordance with clinical improvement.
• The task was reliable and well tolerated.
• The task is suitable for FMRI use and may aid dyspnoea neuroimaging research.

## 1 Introduction

The role of cognitive and emotional factors in dyspnoea is supported by a growing body of literature (Hayen et al., 2013), including a consensus statement published by the American College of Chest Physicians (Mahler et al., 2010). For example, anxiety has been shown to exacerbate dyspnoea in patients with asthma (De Peuter et al., 2008, Janssens et al., 2009, Spinhoven et al., 1997) and chronic obstructive pulmonary disease (COPD) (Giardino et al., 2010, Livermore et al., 2008, von Leupoldt and Dahme, 2007). Dyspnoea is the primary debilitating symptom (Herigstad et al., 2011) and a better predictor of mortality than spirometry in COPD (Nishimura et al., 2002). Therefore, understanding brain mechanisms underlying emotional factors of dyspnoea is of great importance, because it represents a potential therapeutic target.

Repeated associations between fear and day-to-day activities associated with dyspnoea may cause heightened anxiety in COPD patients even before the activity starts. For example, a ringing telephone might become associated with the need to get up quickly, resulting in worsening dyspnoea. Over time the association between dyspnoea-inducing physical activity and the ringing telephone may induce anxiety even prior to commencement of physical activity. Such associations between symptoms and emotions may be studied through presentation of symptom-specific cues. Cues can be images or words related to situations associated with the symptom, and can cause the patient to recall feelings connected with the episodes when the symptom was triggered. This approach has been used to elucidate emotional processing and brain mechanisms in a wide range of disorders, including panic disorder (Wittmann et al., 2011), anxiety and phobias (Etkin and Wager, 2007) and pain (Fairhurst et al., 2012, Jackson et al., 2006, Ogino et al., 2007).

In neuroimaging research (e.g. functional magnetic resonance imaging (FMRI)), multiple presentations of stimuli are necessary to generate reliable statistical maps of brain activation. When a stimulus is paired with behavioural data (e.g. visual analogue scale (VAS) ratings, reaction times), brain signal change may be correlated with individual behavioural scores. Varying stimulus strength may allow scaling of responses. This allows robust interpretation of brain activation and may offer insights into mechanisms of stimulus processing pathways. Therefore, a task comprising multiple presentations of symptom-specific cues of varying valence alongside subjective VAS ratings may offer behavioural and mechanistic insight that is presently not obtainable using questionnaires.

The aim of this study was to develop a task based on a set of cues encompassing common activities and situations that evokes the full range of recalled dyspnoea responses in people with COPD. The task was developed for use in neuroimaging to interrogate brain mechanisms underlying emotional processing of dyspnoea recall. To our knowledge, no such task has yet been developed for dyspnoea.

## 2 Methods

### 2.1 Participants

Patients with COPD were recruited from the pulmonary rehabilitation services run by Oxfordshire Primary Care Trust, Oxford Health NHS Foundation Trust and Oxford University Hospitals NHS Trust. The pulmonary rehabilitation course was outpatient based and included two visits, two hours each, per week for six weeks. The research was approved by the Oxfordshire Research Ethics Committe A. The work was done in four stages: stage 1: relevance assessment of a pool of potential stimuli; stage 2: refinement in a larger subject group; stage 3: testing in an FMRI study in patients and healthy controls; and stage 4: final sensitivity testing and correlations with validated questionnaires. Inclusion criteria were diagnosis of COPD and admittance to pulmonary rehabilitation (patients only). Exclusion criteria were diabetes, major psychiatric disorder under treatment, stroke, opioid treatment or oxygen therapy (all stages), plus contraindications to MRI (stages 3 and 4).

### 2.2 Task format

The computerised task (MATLAB, Mathworks Inc, USA; Cogent toolbox (http://www.vislab.ucl.ac.uk/cogent.php)) consisted of dyspnoea-related and neutral word cues presented on a screen for 7 s, followed by two rating scales (VAS, 7 s each, range 0–100% of scale). The first VAS was headed ‘How breathless would this make you feel?’ and the second ‘How anxious would this make you feel?’ Anchors were ‘Not at all’ and ‘Very much’ with the marker always initially appearing at the centre of the scale. Ratings and keystrokes were recorded throughout the task. An upper anchor of low magnitude was deliberately chosen as patients might have previous experiences of life-threatening exacerbations but the focus of the task was on day-to-day dyspnoea.

### 2.3 Pre-testing: generation of word cues

A set of 30 cues was identified through discussions with respiratory practitioners, academics (at the Breathlessness Research Interest Group meeting, 2009) and physiotherapists, all directly involved with the study or treatment of COPD. These groups were asked to identify cues that reflected situations in which patients report breathlessness (e.g. ‘climbing stairs’) based on their own experience and expertise. Word-based cues were chosen as these could be matched for visual stimulation, which eases FMRI analysis. Neutral cues (e.g. ‘listening to music’) were chosen and added by the researchers to ensure that ratings spanned the entire range from no to maximum dyspnoea/anxiety.

### 2.4 Stage 1: testing of relevance

Patients with COPD (*N* = 11, 2F, 68.5 ± 8.7 years) were given the cues as a paper VAS questionnaire (range 0–10 cm, as described above). The purpose of this test was to assess the relevance of the cues for patients with COPD. Patients were encouraged to contribute suggestions for new cues at the end of the questionnaire, and to discuss the relevance of each cue with the experimenter following the completion of the questionnaire.

During the revision process, care was taken to allow for a good distribution of ratings along the scale and exclude confusing or not applicable cues. For example, several patients had previously enjoyed gardening but stopped due to dyspnoea. As the purpose of the task was to evoke responses associated with current dyspnoea levels rather than earlier experiences, which could pre-date diagnosis, such cues were excluded. Other items were changed as patients reported that related or similar items were considered more challenging (e.g. getting dressed versus getting undressed), and that being presented with the less relevant cue was confusing and/or distracting. Some activities were not relevant or only relevant for a subgroup of patients (e.g. housework). Cue relevance was assessed on the basis of (encouraged) verbal feedback.

During this and all subsequent iterations of the study, the following additional questionnaire scores were collected to characterise the sample: Dyspnoea-12 (Yorke et al., 2010), centre for epidemiological studies depression questionnaire (CES-D, (Radloff, 1977)), state and trait anxiety inventory (STAI, (Spielberger et al., 1983)), catastrophic thinking in asthma scale (De Peuter et al., 2008) and pain awareness and vigilance scale (McCracken, 1997). The two latter were modified by substituting ‘breathlessness’ for the words ‘asthma’ and ‘pain’ (catastrophic thinking in dyspnoea scale and dyspnoea awareness and vigilance scale, respectively).

### 2.5 Stage 2: testing of relevance in a larger group

Following adjustments, the revised set (27 cues) was tested in a larger patient group (*N* = 18, 9F, 69.9 ± 7.3 years). Participants visited the laboratory on one occasion, before beginning their pulmonary rehabilitation course. The task was computerised (see task format) and completed in a seated position in a quiet environment with no experimenter input. Training sessions using previously-discarded cues were conducted until the participant could reliably complete the task on their own. Feedback was encouraged after the task.

### 2.6 Stage 3: application of task to the FMRI environment

The final set of words (24 cues) was tested in patients (*N* = 41, 15F, 68.0 ± 8.2) prior to starting a course of pulmonary rehabilitation and healthy age-matched controls (*N* = 40, 16F, 69.1 ± 8.1). FMRI data was obtained using the task and has been published elsewhere (Herigstad et al., 2015).

### 2.7 Stage 4: testing clinical sensitivity

A subset of the patients from stage 3 (*N* = 34, 9F, 68.2 ± 8.9) returned for a second experimental day after successfully completing their 6-week course of pulmonary rehabilitation. Final analysis was done in this paired sample. Sensitivity to clinical change was assessed by comparing post-pulmonary rehabilitation changes in average cue-based ratings to changes in Dyspnoea-12, catastrophic thinking about dyspnoea, St. George’s respiratory questionnaire (SGRQ) and dyspnoea awareness and vigilance.

### 2.8 Data analysis

All behavioural measures were scored by their respective manuals. Data processing was conducted in MATLAB, statistical analysis in SPSS Statistics version 22 (IBM). Average VAS ratings for each subject were correlated with questionnaire scores. We used Cronbach’s alpha to assess internal task reliability, and skewness and kurtosis to interrogate distribution of ratings. Correlations were evaluated with Pearson coefficients and corrected for multiple comparisons (Bonferroni).

## 3 Results

Behavioural and physiological details of all participants are listed in Table 1.

### 3.1 Stage 1

The initial 30 dyspnoea-related cues and their associated ratings (mean% of max) are listed in Table 2. Nine cues were removed for being unclear or not applicable, and 6 cues modified for clarity or relevance. Six new words were added following patients’ suggestions.

### 3.2 Stage 2

Twenty-seven cues were tested in a second group of patients. Following the second stage, 6 cues were modified and 4 removed (Table 3). Based on patients’ suggestions, one item (‘putting on shoes’) was included, hence increasing the total number to 24. Some cues were revised without altering the meaning to gain uniform word lengths to ensure optimal matching for visual stimulation (final average length 13.6 ± 2.6 letters).

### 3.3 Stages 3 and 4

#### 3.3.1 FMRI applicability

**Fig. 1:**
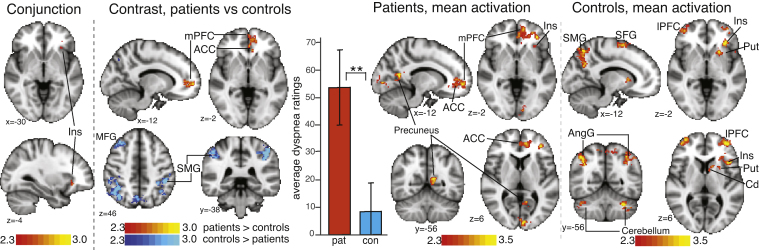
Brain responses to dyspnoea word cues in patients and matched controls (reproduced with permission from Herigstad et al., 2015). Activation (contrasts and conjunction) correlating with visual analogue scale ratings to dyspnoea word cues. Maps are whole-brain analysis, cluster level corrected for multiple comparisons at *p* < 0.05. Maps represent conjunction analysis (activations common to both groups) and comparisons between groups, (patients > controls in red–yellow, controls > patients in blue–lightblue), and mean activations in patients and controls. Bar graph is average ± SD dyspnoea ratings for each group. mPFC (medial prefrontal cortex), ACC (anterior cingulate cortex), Ins (insula), Pcun (precuneus cortex), SMG (supramarginal gyrus), SFG (superior frontal gyrus), ACC (anterior cingulate cortex), AngG (angular gyrus), cerebellum (CrusI and VI) (For interpretation of the references to colour in this figure legend, the reader is referred to the web version of this article.).

During FMRI, the task was split into two sessions administered back-to-back (∼8 min each), which allowed participants a break half-way through the scan. None of the participants asked to stop nor reported difficulties with the task. Patients missed 0.5% of all ratings both before and after pulmonary rehabilitation. Controls missed 0.6% of all ratings. Fig. 1 shows a reproduction of FMRI activation observed using the task (Herigstad et al., 2015).

#### 3.3.2 Reliability

**Fig. 2:**
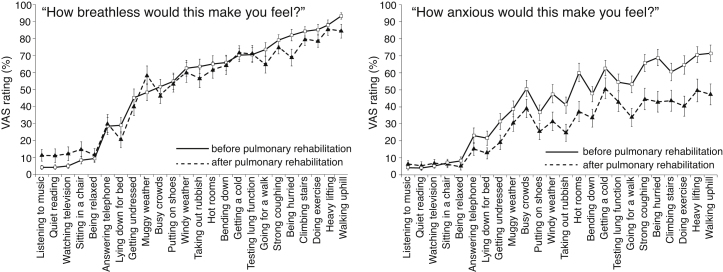
Final set of cues, third iteration (COPD patients only). Average ratings before (closed squares) and after pulmonary rehabilitation (open circles) with standard error. PR (pulmonary rehabilitation).

Reliability results are summarised in Table 4. Before undertaking pulmonary rehabilitation, patients rated the cues for dyspnoea (average 52% ± 29) and anxiety (average 39% ± 22). Following pulmonary rehabilitation, the patients rated cues lower for both dyspnoea (average 49% ± 26) and anxiety (average 28% ± 15). A paired *t*-test shows the following differences in pre- and post-treatment ratings: dyspnoea (*p* = 0.14, NS), anxiety (*p* = 0.0013). Cronbach’s alpha was high for both ratings before and after pulmonary rehabilitation (ranging from 0.90 to 0.96, Table 4), suggesting high internal reliability. Skewness and kurtosis suggests normal distribution for both ratings (Table 4). No ceiling or floor effects were found for either dyspnoea (0% maximum and 0% minimum scores) or anxiety ratings (0% maximum and 2.9% minimum scores) in patients. Fig. 2 shows ratings before and after pulmonary rehabilitation.

#### 3.3.3 Convergence assessments

Ratings before pulmonary rehabilitation correlated with validated respiratory questionnaires and their component scores (Table 5). Only the SGRQ symptom component score did not correlate significantly with VAS scores after correcting for multiple comparisons.

Reduction in anxiety cue ratings following pulmonary rehabilitation correlated with falls in questionnaire scores. Fall in anxiety correlated with fall in Dyspnoea-12 score (*r* = 0.51, *P* = 0.002) and its affective component (*r* = 0.59, *P* = 0.0002), with fall in SGRQ score (*r* = 0.62, *P *< 0.0001) and its impact component (*r* = 0.63, *P *< 0.0001), and with fall in catastrophic thinking about dyspnoea score (*r* = 0.57, *P* = 0.0004) and its helplessness (*r* = 0.51, *P* = 0.002) and rumination components (*r* = 0.59, *P* = 0.0003). Change in dyspnoea ratings did not correlate with changes in any questionnaire scores.

#### 3.3.4 Discriminant validity assessments

Controls rated cues lower than patients (dyspnoea mean rating 8% ± 9, *p* < 0.0001; anxiety mean rating 6% ± 5, *p* < 0.0001). Internal consistency was high. Skewness and kurtosis were high, suggesting a flat distribution with positive skew (long tails to the right). Floor effects were found: dyspnoea (0% maximum and 7.5% minimum scores); dyspnoea-related anxiety (0% maximum and 32.5% minimum scores).

## 4 Discussion

In this paper, we describe the development of a computerised cue-based task for exploring recall of dyspnoea and dyspnoea-related anxiety in patients with COPD. Patients tolerated the task well and used the full range of ratings available. Ratings agreed with established measures of dyspnoea and changed in accordance with clinical improvement.

### 4.1 Sensitivity and comparisons with validated dyspnoea measures

The averaged results from the computerised task showed good correlation with established dyspnoea questionnaires. Patients tolerated the task well and used the full range of the scale, suggesting that the task is suitable for neuroimaging research (see Herigstad et al., 2015 for FMRI findings). The power of a computerised task is that each repeat may be combined with brain recordings (e.g. FMRI, magnetoencephalography), thus directly linking brain mechanisms with behaviour or perception. Furthermore, using computerised tasks may help combatting questionnaire fatigue and cues can be adapted for other types of tests (e.g. emotional Stroop tasks).

The task showed high internal consistency (large Cronbach’s alpha), a measure which typically increases with the number of items on the scale. This suggests that some cues may be interrogating similar constructs. While the task was optimised for neuroimaging, where high numbers of trials/repeats are desirable, it could be shortened for other purposes by removing similar items. This would help reduce both task duration and homogeneity.

The task also showed sensitivity to clinical change, specifically to pulmonary rehabilitation. Pulmonary rehabilitation is a programme which reduces dyspnoea (Lacasse et al., 2007) and, to a greater extent, dyspnoea-related anxiety in COPD (Carrieri-Kohlman et al., 2001, Wadell et al., 2013). Average dyspnoea ratings did not change following pulmonary rehabilitation, whereas average dyspnoea–anxiety ratings were significantly reduced. This is in line with the literature, which shows that the affective dimension of dyspnoea is most improved by pulmonary rehabilitation (Wadell et al., 2013). The decrease in anxiety ratings was strongly correlated with falls in other dyspnoea measures, particularly those associated with the affective dimension. Our cue-based task may therefore be a useful complement to existing assessments of emotional components of COPD.

There are numerous tools for dyspnoea assessment, including the Dyspnoea-12 scale (Yorke et al., 2010) and the multidimensional dyspnoea profile (Meek et al., 2012). Our task complements these by using multiple cues and a continuous scale in place of discrete response categories. This optimises its incorporation into neuroimaging research as continuous scales may provide a higher sensitivity to change as individuals are not made to choose one category over another.

### 4.2 Dyspnoea and MRI

The study of dyspnoea is difficult in the MRI environment, although several studies in healthy volunteers have done so successfully (e.g. Banzett et al., 2008, Evans et al., 2002). First, experimentally induced dyspnoea may alter the FMRI signal (e.g. with hypercapnia and/or resistive respiratory loading (Hayen et al., 2012, Hayen et al., 2015)), particularly at higher field strengths (Faull et al., 2015). Second, induced dyspnoea may be difficult to tolerate, especially for a supine COPD patient in a claustrophobic MRI environment. Orthopnoea and lung abnormalities in patients may further complicate both delivery of respiratory stimuli and monitoring of physiological parameters. For dyspnoea research focusing on emotional associations with dyspnoea and anxiety, a cue-based task can circumvent such challenges. It may thus be a useful tool and an important step for investigating recalled emotional components of dyspnoea in clinical populations.

### 4.3 Use in healthy controls

Despite observing floor effects for the task in healthy controls, making it a poor estimate of absolute dyspnoea in this group, there was still sufficient range to give meaningful FMRI results (Fig. 1).

### 4.4 Conclusions

We have devised a task for measuring recall of dyspnoea and dyspnoea-related anxiety that is sensitive to clinical change in COPD and can be used in an FMRI environment. This task and others like it may help improve our understanding of brain mechanisms in chronic dyspnoea.

## Figures and Tables

**Table 1:** Participant details.

	COPD patients (stages)				Controls
	1st	2nd	3rd		3rd
	(mid-PR)	(pre-PR)	(pre-PR)	(post-PR)	(no PR)
*N*	11	18	34	34	40
Age (yrs)	68.8 (8.7)	69.9 (7.3)	68.2 (8.9)	68.2 (8.9)	69.1 (8.1)
Sex (F)	2	9	9	9	16
MRC (1–5)	n/a	3[1]	3[1]	3[1]	1[0]***
SaO_2_ (%)	n/a	94.2 (1.8)	94.6 (2.6)	94.5 (3.1)	96.4 (1.3)**
HR (bpm)	n/a	83.8 (15.8)	80.8 (12.8)	80.9 (13.8)	72.2 (11.0)***
D12	18.3 (10.8)	15.2 (9.6)	11.4 (9.2)	7.8 (6.4)*	0.0 (0.0)***
CES-D	16.2 (9.9)	18.5 (11.0)	13.0 (8.8)	11.5 (7.3)	7.2 (6.6)**
State anxiety	40.9 (9.9)	36.6 (11.4)	34.5 (8.9)	32.5 (9.2)	25.5 (7.3)***
Trait anxiety	35.9 (10.3)	37.1 (9.1)	35.6 (8.8)	32.1 (8.5)**	29.8 (6.8)***
CTS^a^	17.0 (16.5)	14.0 (10.4)	11.0 (10.4)	7.1 (5.2)**	0.0 (0.2)***
AV^b^	49.7 (21.0)	40.2 (16.8)	38.8 (13.5)	37.4 (14.4)	12.9 (11.3)***
SGRQ	n/a	n/a	49.1 (17.3)	42.0 (14.0)***	6.5 (4.6)***

Mean results and standard deviations for all groups, except for MRC score which is median and interquartile range. Abbreviations: PR, pulmonary rehabilitation; MRC score, Medical Research Council breathlessness score; SaO_2_, blood oxygen saturation; HR, heart rate; D12, Dyspnoea-12 questionnaire; CES-D, center for epidemiologic studies depression scale.​

a Modified from use in asthma.

b Modified from use in pain.

* *p* < 0.05.

** *p* < 0.01.

*** *p* < 0.001.

**Table 2:** List of cues, 1st stage.

	VAS item	Modified	Breathless	Anxious	NR
1	Walking uphill		71 (25)	51 (34)	0
2	Climbing stairs		61 (30)	50 (36)	0
3	Cycling on exercise bike		60 (31)	36 (31)	0
4	Walking on treadmill		60 (29)	50 (37)	0
5	Bending down		60 (27)	56 (33)	0
6	Exercising		57 (30)	47 (28)	0
7	Housework		56 (30)	45 (34)	0
8	Getting a cold		55 (36)	49 (37)	1
9	Going shopping		53 (28)	43 (35)	0
10	Lifting heavy bags	Heavy lifting	52 (35)	40 (41)	0
11	Coughing		51 (34)	47 (37)	0
12	Hot rooms		50 (32)	50 (37)	0
13	Walking	Going for a walk	50 (31)	43 (38)	0
14	Windy weather		47 (36)	53 (37)	0
15	Stretching exercises		45 (31)	38 (26)	0
16	Running errands	Rushing	43 (30)	40 (37)	1
17	Taking a bath		41 (36)	34 (35)	2
18	Gardening		40 (36)	32 (32)	0
19	Lifting dumbbells		40 (33)	47 (31)	0
20	Getting dressed	Getting undressed	39 (32)	40 (36)	0
21	Busy crowds		37 (33)	52 (36)	1
22	Sneezing		34 (28)	31 (31)	0
23	Taking a shower		34 (27)	39 (36)	0
24	Getting out of bed	Lying down for bed	33 (29)	39 (35)	0
25	Cooking food		23 (31)	27 (37)	1
26	Standing up		22 (27)	30 (34)	0
27	Doing laundry		21 (30)	24 (29)	3
28	Reading a book	Reading	17 (21)	18 (29)	2
29	Answering telephone		9 (12)	11 (13)	1
30	Listening to music		6 (12)	4 (3)	1
	Average		42	39	
	SD		16	13	

Dyspnoea-related cues for the first patient group, average ratings (% of max) and standard deviation, number of times item was not rated (NR). Items are presented with highest breathlessness rating first, for convenience. Grey squares: removed as not applicable or confusing. Words that were altered are shown together with the new, modified item.

**Table 3:** List of cues, 2nd stage.

	VAS item	Modified	Breathless	Anxious	NR
1	Walking uphill		95 (7)	76 (24)	1
2	Rushing	Being rushed	90 (12)	69 (26)	2
3	Exercising	Doing exercise	88 (11)	63 (26)	2
4	Walking on treadmill		86 (12)	65 (28)	0
5	Heavy lifting		85 (15)	55 (33)	1
6	Climbing stairs		84 (20)	52 (36)	3
7	Cycling on exercise bike		81 (23)	52 (32)	1
8	Lifting dumbbells		78 (15)	67 (25)	0
9	Testing lung function		78 (28)	55 (31)	1
10	Hot rooms		76 (23)	64 (31)	3
11	Coughing	Strong coughing	74 (23)	57 (35)	3
12	Stretching exercises		73 (27)	58 (32)	2
13	Hot, humid weather	Muggy weather	71 (18)	50 (36)	0
14	Bending down		71 (31)	60 (35)	0
15	Getting a cold		68 (27)	53 (34)	0
16	Going for a walk		61 (27)	43 (36)	0
17	Busy crowds		60 (35)	51 (39)	1
18	Windy weather		59 (32)	55 (34)	2
19	Taking out rubbish		52 (39)	37 (40)	0
20	Getting undressed		44 (34)	25 (31)	0
21	Lying down for bed		25 (32)	23 (32)	2
22	Relaxing	Being relaxed	16 (30)	1 (2)	2
23	Answering telephone		13 (26)	7.5 (14)	0
24	Sitting in a chair		11 (18)	8 (17)	0
25	Listening to music		8.6 (15)	5 (17)	0
26	Watching television		7.7 (38)	3 (6)	0
27	Reading	Quiet reading	3 (9)	0 (1)	2
	Average		58	48	
	SD		30	20	

Dyspnoea-related cues for the second patient group, average ratings (% of max) and standard deviation, number of times item was not rated (NR). Items are presented with highest breathlessness rating first, for convenience. Grey squares: removed as not applicable or confusing. Words that were altered are shown together with the new, modified item.

**Table 4:** Reliability of test.

Stage of testing	D/A	Range	Cronbach’s alpha	Skewness	Kurtosis
1^st^	D	6–71%	0.95	0.14	0.68
A	4–56%	0.98	0.07	0.04
2^nd^	D	3–95%	0.86	−1.22	2.83
A	0–78%	0.91	−0.39	−0.10
3^rd^, pre-PR	D	3–92%	0.90	−0.53	0.02
A	3–68%	0.94	−0.30	−0.19
3^rd^, post-PR	D	10–83%	0.95	−0.20	−0.37
A	4–47%	0.96	0.12	−1.56
3^rd^, controls	D	0–30%	0.93	2.67	9.30
A	0–16%	0.97	3.68	15.15

Reliability of test iterations. Abbreviations: D (dyspnoea rating), A (anxiety rating), PR (pulmonary rehabilitation). Kurtosis (coefficient of excess).

**Table 5:** Convergence with validated questionnaires.

Questionnaires		Dyspnoea VAS ratings		Anxiety VAS ratings	
		*r*	*P*	*r*	*P*
Dyspnoea-12	Total	0.51	0.002	0.54	0.001
Physical	0.50	0.003	0.52	0.002
Affective	0.47	0.005	0.51	0.002

SGRQ	Total	0.80	<0.0001	0.76	<0.0001
Symptom	0.41	0.015 (NS)	0.42	0.014 (NS)
Activity	0.70	<0.0001	0.61	<0.0001
Impact	0.73	<0.0001	0.75	<0.0001

Convergent validity assessments. Abbreviations: VAS, visual analogue scale; SGRQ, St. George’s respiratory questionnaire. *r* = Pearson’s correlation coefficient. Bonferroni correction assumes acceptable *P* level at 0.0071.
